# Aflatoxigenic potential of *Aspergillus quadrilineatus*: implications for food safety in arid climates

**DOI:** 10.3389/fmicb.2026.1724920

**Published:** 2026-04-30

**Authors:** Fahad A. Alkhayyat, Nisha Vadakkancheril Somasekharan

**Affiliations:** 1 Department of Biological Sciences, Kuwait University, Kuwait City, Kuwait; 2 Department of Biological Sciences, Kuwait University College of Science, Khaldiya, Kuwait

**Keywords:** aflatoxin, arid and semi-arid climate, *Aspergillus quadrilineatus*, food safety, fungi, Kuwait, mycotoxin

## Abstract

Mycotoxins produced by *Aspergillus* species pose a major threat to food safety, particularly in warm and arid regions where environmental conditions favor fungal persistence and toxin biosynthesis. *Aspergillus quadrilineatus*, a member of *Aspergillus* section *Nidulantes*, is a documented producer of sterigmatocystin, the penultimate precursor of aflatoxin biosynthesis, yet its food-colonization potential remains poorly characterized. In this study, we investigated the growth characteristics, sporulation capacity, and mycotoxigenic potential of nine *A. quadrilineatus* isolates recovered from agricultural soils, compost, and poultry farm environments in Kuwait. Species identity was confirmed by morphological examination and ITS sequencing. All isolates exhibited optimal growth between 35 and 40°C, with several strains retaining growth above 45°C, indicating pronounced thermotolerance. All strains successfully colonized peanuts and strawberries under experimental conditions and produced high conidial loads. Sterigmatocystin was detected in all isolates, with maximal production observed on day 7 of incubation. High-performance liquid chromatography analysis demonstrated that multiple isolates produced substantial quantities of aflatoxins B^1^, B^2^, G^1^, and G^2^ on infected food commodities. In peanuts, total aflatoxin concentrations reached 7.38, 6.51, and 6.32 ppm in the highest-producing strains, exceeding international regulatory limits by several orders of magnitude. In strawberries, aflatoxin production was generally lower but remained significant, with G-type aflatoxins frequently predominating. Collectively, these results demonstrate that indigenous *A. quadrilineatus* strains can survive arid climatic conditions, infect food commodities, and contaminate them with carcinogenic mycotoxins, identifying this species as an under-recognized food safety risk in arid and semi-arid regions.

## 1 Introduction and objectives

Mycotoxins are toxic secondary metabolites produced by a wide range of filamentous fungi that frequently contaminate food and feed commodities worldwide. Their presence in the food chain represents a major concern for public health, animal health, and global food security, particularly in regions characterized by warm climates and suboptimal storage conditions (Vining, 1990; Keller et al., 2005). Unlike many microbial contaminants, mycotoxins are chemically stable, resistant to thermal processing, and often persist during food preparation and storage, thereby increasing the risk of chronic exposure (Midio et al., 2001; Moss, 2002).

Among mycotoxigenic fungi, species belonging to the genus *Aspergillus* are of particular importance due to their ecological ubiquity, metabolic versatility, and ability to colonize a wide variety of substrates. Members of this genus are responsible for the biosynthesis of some of the most potent naturally occurring toxins, including aflatoxins, which are strongly hepatotoxic, immunosuppressive, and carcinogenic (Moss, 2003; Baranyi et al., 2015). Aflatoxin B^1^ is classified as a Group 1 human carcinogen and is considered the most powerful naturally produced carcinogen known to date (Moss, 2002; Baranyi et al., 2015). Consequently, contamination of agricultural commodities with aflatoxins results in substantial economic losses and stringent regulatory controls worldwide.

Sterigmatocystin (ST) is the penultimate intermediate in the aflatoxin biosynthetic pathway and is itself a toxic and carcinogenic compound (Brown et al., 1996; Rank et al., 2011). Although historically considered less relevant than aflatoxins, sterigmatocystin has gained increasing attention due to its frequent detection in food products and its association with species outside the classical aflatoxigenic groups (Rank et al., 2011; Frisvad et al., 2011). Importantly, the presence of sterigmatocystin often signals the genetic and metabolic potential for aflatoxin biosynthesis under favorable conditions (Keller et al., 1997; Amaike and Keller, 2009).

*Aspergillus quadrilineatus* is a soil-borne filamentous fungus belonging to *Aspergillus* section *Nidulantes* (formerly *Emericella*). Species within this section are characterized by a sexual reproductive stage and are widely distributed in terrestrial environments, including agricultural soils, compost, and animal-associated substrates (Samson et al., 2014; Chen et al., 2016). *A. quadrilineatus* has been reported as a producer of sterigmatocystin and, under certain conditions, aflatoxins (Gbodi, 1993; Frisvad et al., 2004; Mahata et al., 2022). In addition to its toxigenic capacity, this species has been implicated in opportunistic human infections, including cases of invasive aspergillosis and fungal sinusitis (Polacheck et al., 1992).

Despite its documented toxigenic potential, *A. quadrilineatus* remains poorly studied compared with classical aflatoxin producers such as *A. flavus* and *A. parasiticus*. In particular, its ecological distribution, thermotolerance, and ability to colonize and intoxicate food commodities under arid and semi-arid climatic conditions are largely unexplored. Arid regions, including the Arabian Gulf, present environmental conditions—high temperatures, low water activity, and frequent dust exposure—that may favor the persistence and dissemination of xerotolerant and thermotolerant fungal species (Magan and Aldred, 2007; Baranyi et al., 2015).

Kuwait represents a relevant model for investigating mycotoxigenic fungi in arid environments due to its extreme summer temperatures, reliance on imported food commodities, and expanding local agricultural production. Although regulatory frameworks exist within the Gulf Cooperation Council (GCC) to limit aflatoxin contamination in food products, including strict maximum permissible levels for nuts and dried fruits, data on the environmental reservoirs and indigenous toxigenic fungi contributing to contamination remain scarce (GCC Standardization Organization, 2021; Alamir et al., 2023). In particular, little is known about the occurrence, physiology, and food-colonization capacity of *A. quadrilineatus* in Kuwait.

The present study addresses this knowledge gap by characterizing *A. quadrilineatus* isolates recovered from agricultural soils, compost, and poultry farm environments in Kuwait. We investigate their growth characteristics across a range of temperatures relevant to storage and arid climates, their sporulation capacity on food substrates, and their ability to biosynthesize sterigmatocystin and aflatoxins during food colonization. By using peanuts and strawberries as representative food commodities, this work evaluates the potential of *A. quadrilineatus* to contribute to post-harvest spoilage and mycotoxin contamination under conditions relevant to local markets.

Collectively, this study aims to (i) assess the ecological fitness of *A. quadrilineatus* in arid environments, (ii) determine its mycotoxigenic potential on commonly consumed foods, and (iii) highlight its relevance as an under-recognized food safety risk in Kuwait and similar climatic regions. The findings provide critical baseline data for future surveillance, risk assessment, and mitigation strategies targeting mycotoxin contamination beyond the classical aflatoxigenic species.

## 2 Material and methods

### 2.1 Culture conditions

The culture numbers and sources of the nine *A. quadrilineatus* strains used in this study are listed in Table 1. Stock cultures were maintained on malt extract agar (MEA; Sigma-Aldrich). The cultures were maintained at 4°C for short-term storage, while conidia were preserved at −80°C in glycerol for long-term storage.

**TABLE 1:** *Aspergillus quadrilineatus* isolates included in this study.

Isolate no.	Compost strains
C.46	*Aspergillus quadrilineatus*
C.160	*Aspergillus quadrilineatus*
C.62	*Aspergillus quadrilineatus*
	Agricultural field strains
A.192	*Aspergillus quadrilineatus*
A.61	*Aspergillus quadrilineatus*
A.221	*Aspergillus quadrilineatus*
	Poultry farm strains
P.38	*Aspergillus quadrilineatus*
P.74	*Aspergillus quadrilineatus*
P.19	*Aspergillus quadrilineatus*

### 2.2 DNA Extraction and Identification

Genomic DNA was extracted from 14-day-old pure cultures of *Aspergillus quadrilineatus* grown on malt extract agar at 25°C using the PrepMan Ultra Sample Preparation Reagent (Applied Biosystems, Foster City, CA, United States). Fungal mycelium was suspended in 200 μL of PrepMan Ultra reagent in sterile 1.5 mL Safe-Lock Eppendorf tubes (Hamburg, Germany) and mechanically disrupted using a zirconia bead beater for 30 s at 2,500 beats min ^–1^. Samples were subsequently incubated in a boiling water bath (100°C) for 10 min and centrifuged at 13,000 rpm for 3 min. The supernatant containing genomic DNA was carefully transferred to new sterile microcentrifuge tubes. To eliminate residual spores, samples were centrifuged repeatedly prior to storage at −20°C until PCR amplification.

The internal transcribed spacer (ITS) region was amplified using primers ITS1 (5’-TCCGTAGGTGAATGCGG-3’) and ITS4 (5’-TCCTCCGCTTATTGATATGC-3’) (Cantrell et al., 2006), yielding an amplicon of approximately 550–556 bp. PCR reactions were performed using Ready-To-Go Taq PCR beads (GE Healthcare, Chalfont-St. Giles, United Kingdom) with 1.0 μL of template DNA, 30 pmol of each primer, and molecular-grade water to a final volume of 25 μL. Amplification products were verified by electrophoresis on 1% agarose gels using a genomic DNA ladder (Sigma-Aldrich, St. Louis, MO, United States).

PCR products were purified using the QIAquick PCR Purification Kit (Qiagen, Germantown, MD, United States) and sequenced using the BigDye Terminator v3.1 Cycle Sequencing Kit (Applied Biosystems). Sequencing reactions were purified by ethanol–sodium acetate precipitation, washed with 70% ethanol, air-dried, and resuspended in 20 μL Hi-Di formamide (Applied Biosystems). Samples were denatured and loaded onto MicroAmp optical 96-well plates for capillary electrophoresis using an ABI 3130xl Genetic Analyzer (Applied Biosystems). Sequence data were analyzed using Sequencing Analysis software v5.2 and aligned with reference sequences from GenBank using ClustalX2 (Thompson et al., 1997). Representative sequences were deposited in the NCBI BioSample database under accession number PRJNA1402885.

### 2.3 Microscopy

Each *Aspergillus quadrilineatus* isolate was cultivated on malt extract agar (MEA) and incubated at different temperatures as specified for growth analysis. Following incubation, colony morphology and reproductive structures were examined using light microscopy (LM) and scanning electron microscopy (SEM), as described previously (Matsuzawa et al., 2012).

For SEM analysis, cleistothecia were collected and some were crushed to release ascospores from 4-week-old stock cultures. Ascospore suspensions were directly mounted onto specimen stubs. Samples were air-dried at 25°C, sputter-coated with a gold–palladium alloy, and examined using a field-emission scanning electron microscope (Leo Supra 50P) operated at an accelerating voltage of 10 kV.

### 2.4 Temperature growth profile

The temperature growth profiles of *Aspergillus quadrilineatus* isolates were evaluated on malt extract agar (MEA). A standardized inoculum from each isolate was point-inoculated onto the center of MEA plates, which were then incubated at temperatures ranging from 15 to 50°C. Colony diameter was measured after 2 weeks of incubation, and radial growth was recorded in centimeters (cm). For each isolate, growth at each temperature was assessed based on visible colony development. Trace growth was recorded as (+), whereas absence of visible growth was recorded as (0). These data were used to compare the thermal tolerance and optimal growth range of the tested isolates.

### 2.5 Growth analysis

Fungal biomass was quantified by measuring mycelial dry weight following growth in Borrow’s amino acid (BOA) broth (composition provided in Supplementary Table 1; Borrow et al., 1961) at 35°C. Cultures were harvested on days 2, 3, 4, 7, and 10 of incubation by vacuum filtration through pre-weighed Whatman No. 1 filter paper. Prior to filtration, filter papers were oven-dried to constant weight to eliminate residual moisture and weighed accurately.

Following filtration, the filter papers containing fungal mycelia were dried overnight at 40–50°C in a drying oven. The combined weight of the dried filter paper and fungal biomass was then recorded. Mycelial dry weight was calculated by subtracting the initial weight of the dry filter paper from the final combined weight, as described previously by Yamazaki et al. (2007). Each experiment was conducted in triplicate, and results are presented as mean values from three independent biological replicates.

### 2.6 *In vitro* sterigmatocystin detection by thin layer chromatography

Sterigmatocystin (ST) production was assessed using 10-day-old *Aspergillus quadrilineatus* stock cultures grown on malt extract agar. Toxin induction was performed in BOA broth supplemented with 0.3% (w/v) methyl-β-cyclodextrin and incubated at 25°C, as previously described (Frisvad et al., 2004). A loopful of conidia from each isolate was suspended in 1 mL of 0.001% Triton X-100, and aliquots were used to inoculate individual slants containing 2 mL BOA broth. Cultures were incubated at 35°C.

Sterigmatocystin was extracted by adding 2 mL chloroform to each culture, followed by vortexing for 1 min and allowing phase separation for 5 min. This extraction step was repeated six times to ensure maximal recovery. Combined chloroform extracts were allowed to settle for 30 min and then centrifuged at 5,000 rpm for 15 min. The lower organic phase (approximately 1.5 mL) was carefully transferred to a new vial and evaporated to dryness under ambient conditions. Dried residues were resuspended in 500 μL chloroform.

For thin-layer chromatography (TLC) analysis, 10 μL of each extract was spotted onto silica gel 60 TLC plates (Sigma-Aldrich). Separation was performed using a toluene: ethyl acetate: 90% formic acid (5:4:1, v/v/v) solvent system. Plates were sprayed with 20% (w/v) aluminum chloride in ethanol, baked at 120°C for 5 min, and visualized under long-wave UV light (365 nm) following established protocols (Amaike and Keller, 2009). Sterigmatocystin was identified by comparison with authenticated reference standards (Sigma-Aldrich).

Digital images of TLC plates were captured under identical exposure conditions. Sterigmatocystin production was quantified semi-quantitatively using ImageJ software by measuring integrated pixel density after background subtraction (Schneider et al., 2012). Extractions were performed on days 2, 3, 4, 7, and 10 of incubation, and all samples were analyzed in triplicate.

### 2.7 Seed infections

Mature peanut seeds (*Arachis hypogaea*) were prepared by manually removing the seed coat, separating the two cotyledons, and carefully excising the embryo without damaging the cotyledon tissue. Fresh strawberries (*Fragaria ananassa*) were cut into approximately 1 cm diameter pieces. All plant materials were surface sterilized by immersion in 0.05% (v/v) sodium hypochlorite prepared in sterile distilled water for 3 min, followed by rinsing in sterile distilled water for 30 s. Samples were then briefly immersed in 70% (v/v) ethanol for 5 s and rinsed again in sterile distilled water for 30 s before being drained completely. All procedures were conducted under aseptic conditions.

Surface-sterilized peanut cotyledons and strawberry pieces were inoculated with a conidial suspension adjusted to 1 × 10^5^ spores mL ^–1^. For each treatment, 20 pieces were immersed in 40 mL of sterile distilled water containing fungal conidia in 50 mL centrifuge tubes. Control treatments consisted of plant material immersed in sterile distilled water without spores. Tubes were gently agitated for 30 min at 50 rpm using a rotary shaker to ensure uniform inoculation.

Following inoculation, plant materials were transferred to sterile Petri dishes lined with three layers of moistened sterile filter paper to maintain high relative humidity. Samples were incubated at 35°C for 7 days in the dark. Filter papers were re-moistened daily to prevent desiccation. All treatments were performed in triplicate, and analyses were conducted using equal numbers of seeds or fruit pieces per replicate, as described previously (Kales et al., 2008).

### 2.8 Food item colonization and mycotoxin analysis

#### 2.8.1 Sporulation on infected food substrates

To quantify sporulation on infected food commodities, a peanut seed and a strawberry piece each were inoculated, per treatment, as described above, were transferred into 50 mL Falcon tubes containing 10 mL of sterile distilled water. Samples were vortexed vigorously for 1 min to dislodge conidia from the substrate surface. A 10 μL aliquot from each suspension was used for conidial enumeration with a hemocytometer. Conidia counts were performed in triplicate for each sample.

#### 2.8.2 Mycotoxin extraction from peanut seeds and strawberries

Inoculated peanut seeds and fresh strawberry pieces were collected in 50 mL Falcon tubes containing 5 mL of distilled water and vortexed vigorously for 1 min. Acetone (5 mL) was added to each tube, followed by shaking for 10 min at 150 rpm using a rotary shaker. Samples were allowed to stand at room temperature for 5 min before the addition of 5 mL chloroform. Tubes were shaken again for 10 min at 150 rpm, allowed to stand for an additional 10 min, briefly vortexed, and centrifuged at 2,000 rpm for 15 min to facilitate phase separation.

The lower organic (chloroform) phase was carefully transferred to new tubes and allowed to evaporate to dryness over 3 days. The dried residues were reconstituted by adding 5 mL of 0.1 M NaCl in methanol/water (55:45, v/v) and 2.5 mL hexane, followed by vigorous vortexing for 1 min. Samples were centrifuged at 2,000 rpm for 5 min, and the hexane layer was collected. The remaining aqueous phase was re-extracted with an additional 2.5 mL hexane, and the extraction procedure was repeated. Combined hexane extracts were dried and stored at 4°C until further analysis.

#### 2.8.3 Sterigmatocystin detection by thin-layer chromatography

Dried hexane extracts were resuspended in 500 μL methanol, and 10 μL aliquots were applied to silica gel TLC plates. Chromatographic separation was performed using a toluene: ethyl acetate: 90% formic acid (5:4:1, v/v/v) solvent system. Each treatment was analyzed in triplicate (Kales et al., 2008). Sterigmatocystin spots were visualized as described previously and quantified using ImageJ software by measuring integrated pixel density after background subtraction (Schneider et al., 2012).

To evaluate density-dependent toxin production, sterigmatocystin levels were compared among strains using triplicate measurements. Results are presented as mean values ± standard deviation.

#### 2.8.4 Determination of aflatoxins by HPLC

Aflatoxins B^1^ (AFB1), B^2^ (AFB2), G^1^ (AFG1), and G^2^ (AFG2) were quantified using high-performance liquid chromatography (HPLC) with fluorescence detection. Shimadzu Nexera X2 HPLC, equipped with SPD 20A prominence with UV/V is detected at 365 nm. Separation was done on a Zorbax XDB Eclipse C18, 5 μm, 4.6 × 150 mm column with a flow of 1.5 mL/min using water: methanol: acetonitrile (50:40:10) as mobile phase.

Aflatoxin standards were used for calibration and quantification. A standard solution was analyzed at five concentration levels (0.025, 0.05, 0.10, 0.15, and 0.20 ppm) to construct calibration curves for aflatoxin using the external standard method.

Individual aflatoxins were identified by comparing sample retention times with those of the standard mixture. The observed retention times in the standard chromatogram were approximately 2.44 min for AFG2, 2.78 min for AFG1, 3.34 min for AFB2, and 3.87 min for AFB1.

Calibration curves were generated by plotting peak area against aflatoxin concentration, and linear regression equations were obtained for each analyte using the instrument software. Sample chromatograms were processed by integrating the peaks corresponding to AFB1, AFB2, AFG1, and AFG2, and concentrations were calculated using the respective calibration equations. Each sample was analyzed in duplicate, and results were expressed as mean ± standard deviation (SD). Total aflatoxin content was calculated as the sum of the four individual aflatoxins. Aflatoxins not detected under the analytical conditions were reported as 0.000 ppm.

### 2.9 Statistical analysis

To evaluate whether aflatoxin production by individual strains differed from the overall production pattern, one-sample *t*-tests were performed for each strain × aflatoxin combination. The reference value for each test was the global mean concentration of the corresponding aflatoxin (B^1^, B^2^, G^1^, or G^2^) calculated across all samples and matrices.

Tests were conducted only when variance was present between technical replicates; cases with zero variance were not statistically testable and were excluded from inferential analysis. To control for multiple comparisons, Benjamini–Hochberg false discovery rate (FDR) correction was applied, and results with adjusted *q*-values < 0.05 were considered statistically significant.

All statistical analyses and graphical visualizations were performed using Python, employing standard scientific libraries for data handling, hypothesis testing, and plotting.

## 3 Results

### 3.1 Isolates identification and growth characterization

**FIGURE 1:**
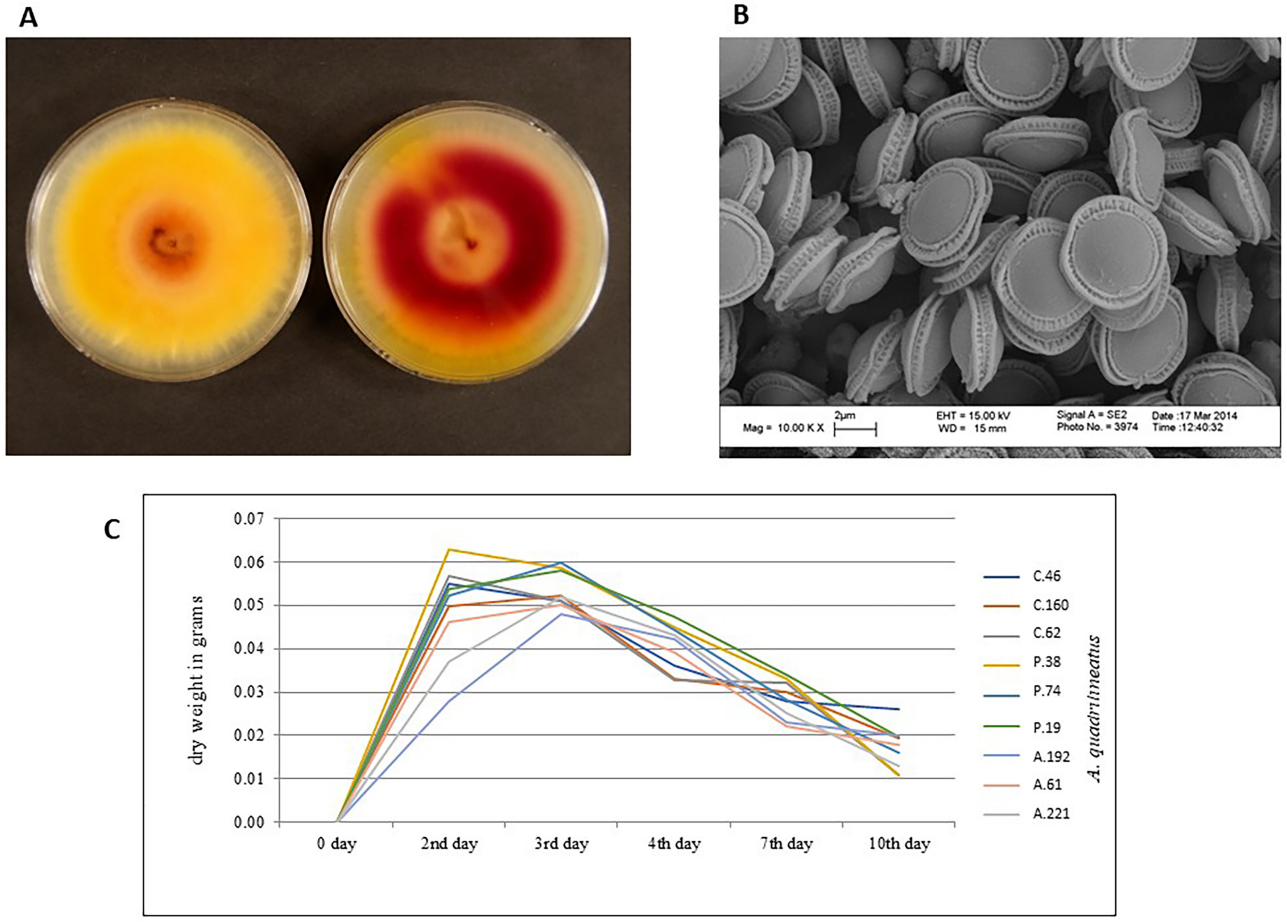
Morphological and growth characteristics of *Aspergillus quadrilineatus*. **(A)** Colony morphology of an *Aspergillus quadrilineatus* isolate grown on malt extract agar (MEA), showing obverse (left) and reverse (right) views. **(B)** Scanning electron microscopy (SEM) image of *A. quadrilineatus* ascospores at 10,000× magnification. **(C)** Growth kinetics of *A. quadrilineatus* isolates over a 10-day incubation period, measured as mycelial dry weight.

A comprehensive survey was conducted to characterize the diversity of *Aspergillus* species isolated from agricultural and livestock-associated environments in Kuwait (unpublished data). Multiple *Aspergillus* clades were identified, of which a single clade, *Aspergillus quadrilineatus*, was selected for detailed characterization. Colony morphology, cleistothecia and ascospores were examined under light microscope and electron microscopy for accurate identification (Figures 1A,B; Supplementary Figure 1). Isolate Identifications were further confirmed by sequencing the ITS region of three isolates.

The cardinal temperature ranges (minimum, optimum, and maximum) for all *A. quadrilineatus* isolates were determined. Optimal growth occurred between 35 and 40°C for all strains (Table 2). Notably, all isolates were capable of growth at temperatures ranging from 20 to 30°C, a range commonly encountered in food storage and domestic environments.

**TABLE 2:** Temperature growth profile of *Aspergillus quadrilineatus* isolates on malt extract agar (MEA).

Strains	Two weeks of growth on MEA media in cm											
	15° C	20° C	25° C	30° C	35° C	40° C	45° C	46° C	47° C	48° C	49° C	50° C
P.19	1	2	3	5.5	7.5	8	6.2	4	3.2	0.5	0	0
P.38	1.3	3.5	5.5	7	8	8.5	8	4.5	2.5	0.5	0	0
P.74	1.5	2	3.3	7	8.5	8	5.5	2.8	2.5	0.5	0	0
C.46	1.5	3	5.5	7	8	8.5	7.3	5.3	2.6	1	+	0
C.160	1	3	6.2	7	8	8.5	6.8	4	3.4	2	+	0
C.62	1.2	3.2	6.2	6.8	7.8	8.5	7.2	3	3	2	0	0
A.61	1	2.8	3.5	6.7	8	8.2	5.8	3	2	1	0	0
A.192	1.3	3	3.5	6.5	8.5	8	5.5	3	2	0.5	0	0
A.221	1	2	2.8	6.5	8.5	8.2	4.2	2.2	1	0.5	0	0

Radial colony growth in centimeter (cm) was measured after 2 weeks of incubation across a temperature range of 15–50°C. Symbols indicate trace growth (+) or no detectable growth (0) where applicable.

Vegetative growth was assessed by measuring mycelial dry weight in liquid BOA medium at 35°C, corresponding to the determined optimal growth temperature (Table 2). All isolates exhibited rapid exponential growth, with maximal mycelial biomass observed on day 2 of incubation (Figure 1C). From day 4 onward, a gradual decline in mycelial mass was observed through the final sampling point on day 10. Consistent with patterns reported for other *Aspergillus* species, pigment accumulation became apparent following the exponential growth phase, suggesting a metabolic shift toward secondary metabolism (Calvo et al., 2002).

Statistical analysis using one-sample *t*-tests indicated significant variation in vegetative biomass among strains, with isolate P.19 producing the highest mycelial mass and A.192 producing the lowest (*p* < 0.05). As vegetative growth is not necessarily predictive of mycotoxin biosynthetic capacity (O’Gorman, 2011), subsequent analyses focused on evaluating the sterigmatocystin-producing potential of *A. quadrilineatus* isolates.

### 3.2 *Aspergillus quadrilineatus* isolates can infect food commodities

**FIGURE 2:**
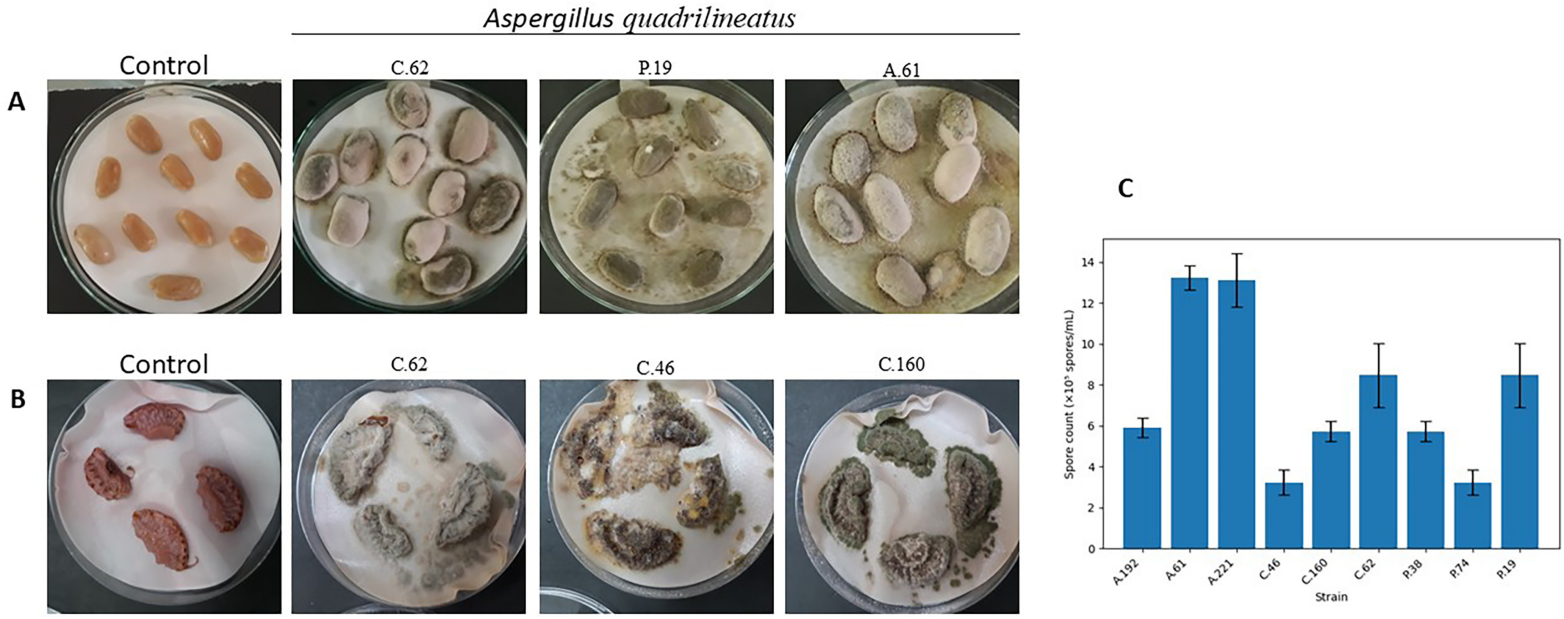
Colonization of food substrates by *Aspergillus quadrilineatus*. **(A)** Visual discoloration of peanut seeds following inoculation with *A. quadrilineatus* isolates after 7 days of incubation. **(B)** Visual discoloration of strawberry samples following inoculation with *A. quadrilineatus* isolates after 7 days of incubation. **(C)** Conidial production on peanut seeds expressed as log-transformed conidia counts per peanut seed.

To test the isolate’s capability to infect food commodities, raw peanuts were collected from Kuwait’s market and infected with the test strains (Figure 2A). Spore production varied markedly among the tested strains isolated from agricultural, compost, and poultry sources. Mean spore counts ranged from 3.23 × 10^5^ to 13.23 × 10^5^ spores/mL per peanut seed, with clear differences in both central tendency and dispersion across isolates (Figure 2C).

Among the agricultural strains, A.61 and A.221 exhibited the highest sporulation levels, with mean spore counts of 13.23 ± 0.60 × 10^5^ and 13.13 ± 1.30 × 10^5^ spores/mL, respectively. In contrast, A.192 showed a substantially lower mean value (5.91 ± 0.48 × 10^5^ spores/mL). Compost-derived strains displayed intermediate variability: C.62 had a relatively high mean spore count (8.47 ± 1.55 × 10^5^ spores/mL), whereas C.46 showed one of the lowest sporulation levels (3.23 ± 0.60 × 10^5^ spores/mL). A similar pattern was observed among poultry strains, where P.19 demonstrated elevated sporulation (8.47 ± 1.55 × 10^5^ spores/mL), while P.74 exhibited low spore production (3.23 ± 0.60 × 10^5^ spores/mL).

One-way analysis of variance confirmed a highly significant difference in spore concentrations among all strains (*p* = 2.36 × 10 ^–10^; Supplementary Table 2). When individual strains were compared against the global mean spore concentration (7.46 × 10^5^ spores/mL) using one-sample *t*-tests, A.61 and A.221 were identified as having significantly higher spore counts (*p* < 0.05), whereas C.62 and P.19 did not differ significantly from the global mean. All remaining strains showed significantly lower spore counts relative to the global mean.

Overall, these results demonstrate pronounced strain-dependent differences in sporulation capacity, with agricultural strains A.61 and A.221 representing the highest spore-producing isolates under the conditions tested.

### 3.3 *Aspergillus quadrilineatus* isolates biosynthesize sterigmatocystin

**FIGURE 3:**
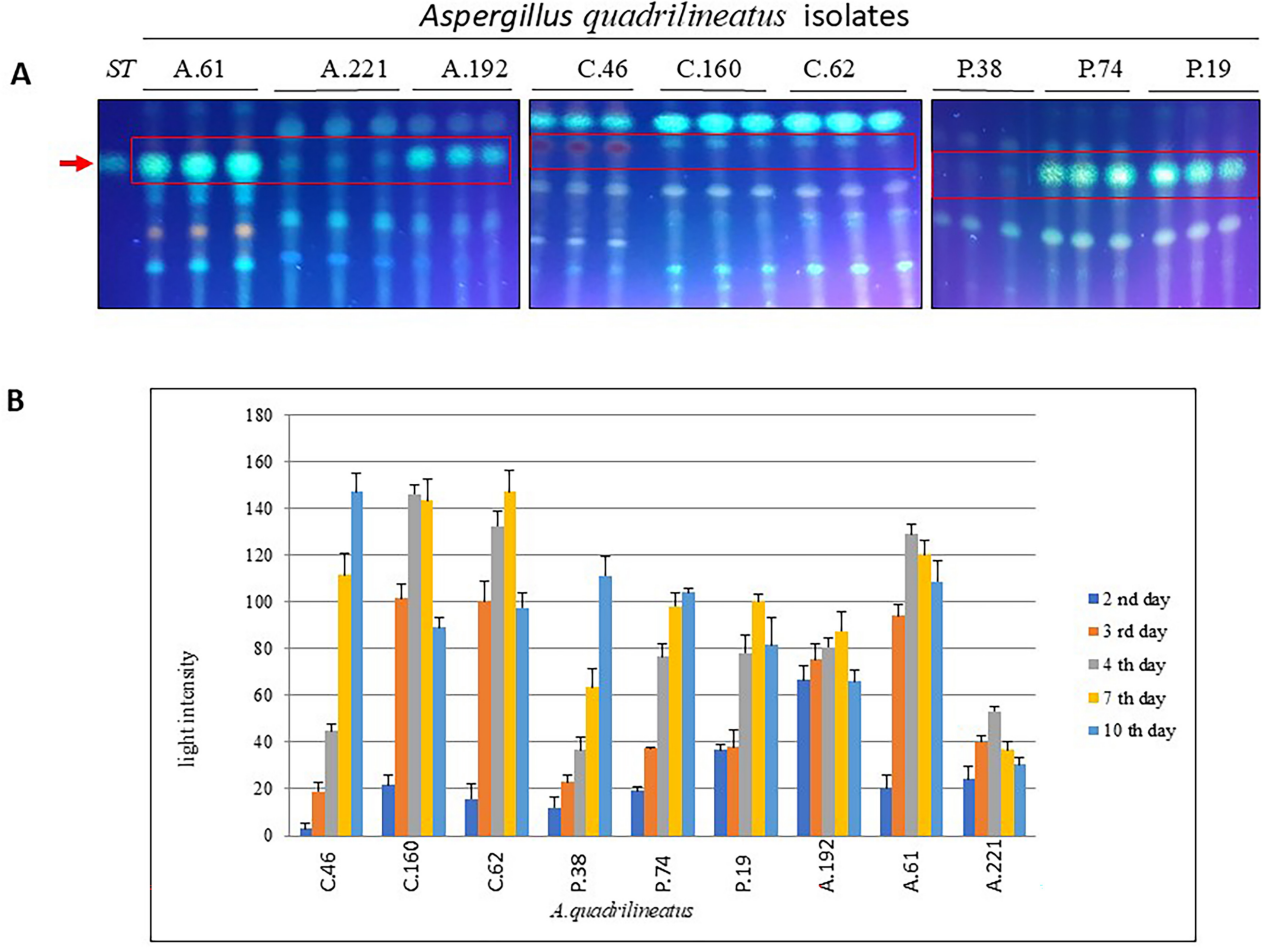
Sterigmatocystin (ST) production by *Aspergillus quadrilineatus* in liquid culture. **(A)** Thin-layer chromatography (TLC) plates showing ST produced by *A. quadrilineatus* strains cultivated in liquid broth medium; the red arrow indicates the ST reference control. **(B)** Quantification of ST production by *A. quadrilineatus* in broth medium at different incubation time points (days), as estimated by ImageJ software (Schneider et al., 2012).

As the penultimate precursor in the aflatoxin biosynthetic pathway, sterigmatocystin (ST) production was monitored to evaluate the mycotoxigenic potential of the tested strains. ST production was assessed using thin-layer chromatography (TLC) at the 2nd, 3rd, 4th, 7th, and 10th days of incubation (Figure 3A). Densitometric quantification of ST spots was performed using ImageJ software by measuring relative light intensities (Figure 3B).

**FIGURE 4:**
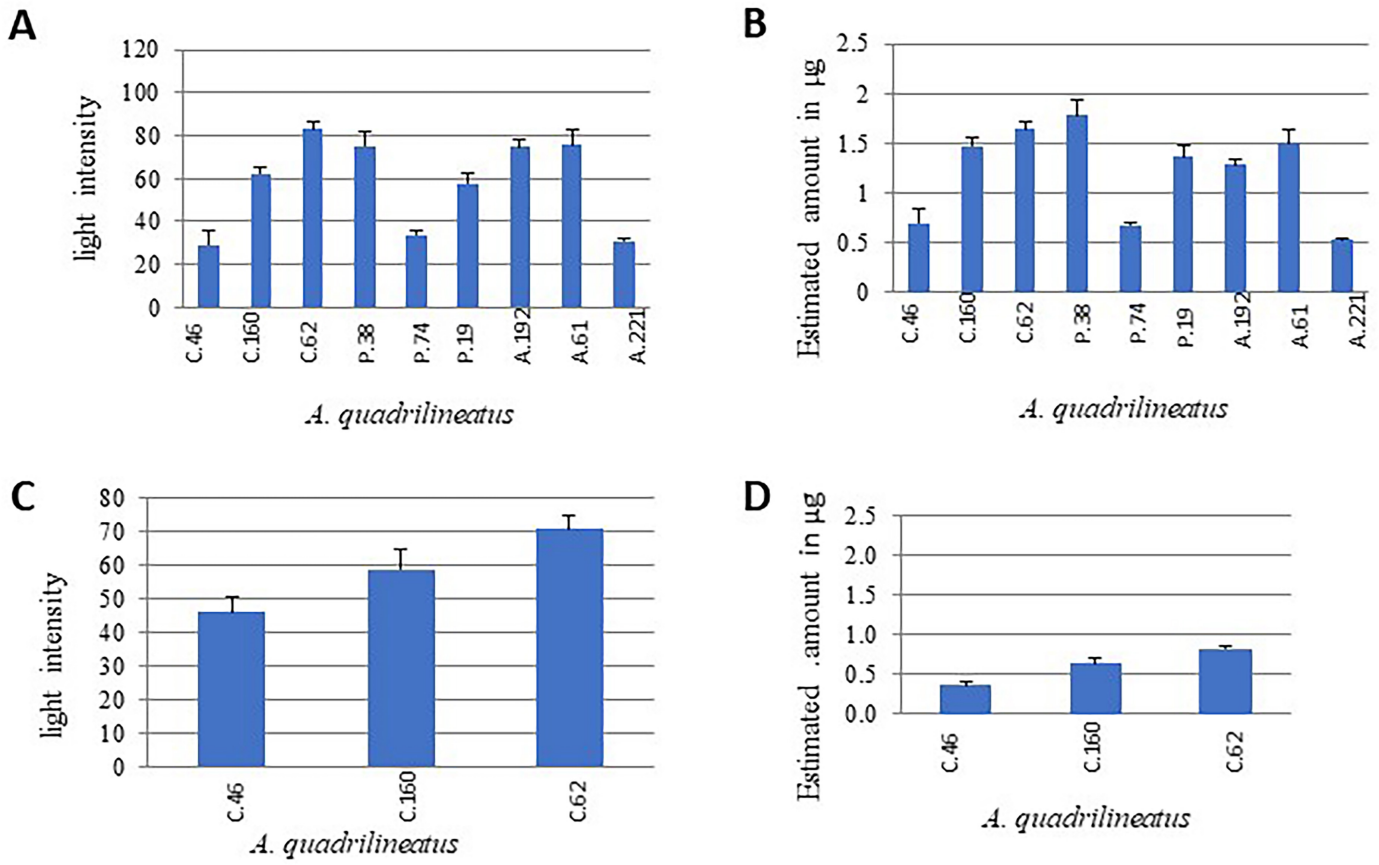
Sterigmatocystin extracted from food commodities. **(A)** Relative light intensity of ST extracted from peanut samples as quantified using ImageJ software. **(B)** Amounts of ST (μg) in peanut samples calculated from light intensity values using the reference control. **(C)** Relative light intensity of ST extracted from strawberry samples quantified using ImageJ software. **(D)** Amounts of ST (μg) in strawberry samples calculated from light intensity values using the reference control (Schneider et al., 2012).

Sterigmatocystin accumulation increased progressively over time, reaching maximal levels on the 7th day of incubation in most strains. Compost isolates (C.160 and C.62) and agricultural isolates (A.61 and A.192) exhibited the highest levels of ST production. In contrast, the agricultural isolate A.221 consistently produced low ST levels throughout the incubation period. The remaining isolates showed comparable intermediate ST production profiles. Representative TLC plates illustrating ST production on the 7th day for all strains, alongside the reference standard, are presented in Figure 4.

Sterigmatocystin production was further evaluated in infected substrates, peanut seeds and fresh strawberries (Figure 2), on the 7th day of incubation. ST was extracted and quantified (μg) by comparing TLC spot intensities with a standard reference using ImageJ software. Six isolates (C.62, C.160, A.61, A.192, P.38, and F19) produced high levels of sterigmatocystin, ranging from approximately 1.0 to 1.5 μg per eight infected peanut seeds (Figures 4A, B). Nevertheless, isolates characterized by lower conidial output, including C.46, N.74, and A.221, were still capable of producing measurable amounts of ST (approximately 0.5 μg).

Given the close association between compost and agricultural production systems, the sterigmatocystin-producing capacity of compost isolates was further examined using strawberries as a representative local produce. Both C.62 and C.160 produced detectable levels of ST by the 7th day of incubation. In contrast, the *Aspergillus quadrilineatus* isolate C.46 produced comparatively lower ST levels. Quantitative analysis revealed that C.62 and C.160 each produced more than 0.5 μg sterigmatocystin per two strawberries (six cut pieces), whereas C.46 produced less than 0.5 μg (Figures 4C, D).

### 3.4 *Aspergillus quadrilineatus* isolates can intoxicate food with aflatoxin

To confirm the strain’s ability to produce aflatoxins, we carried out aflatoxin extraction from infected peanuts and fresh strawberries, followed by HPLC fractionation and detection. Untreated peanut and strawberry samples were used as control.

**FIGURE 5:**
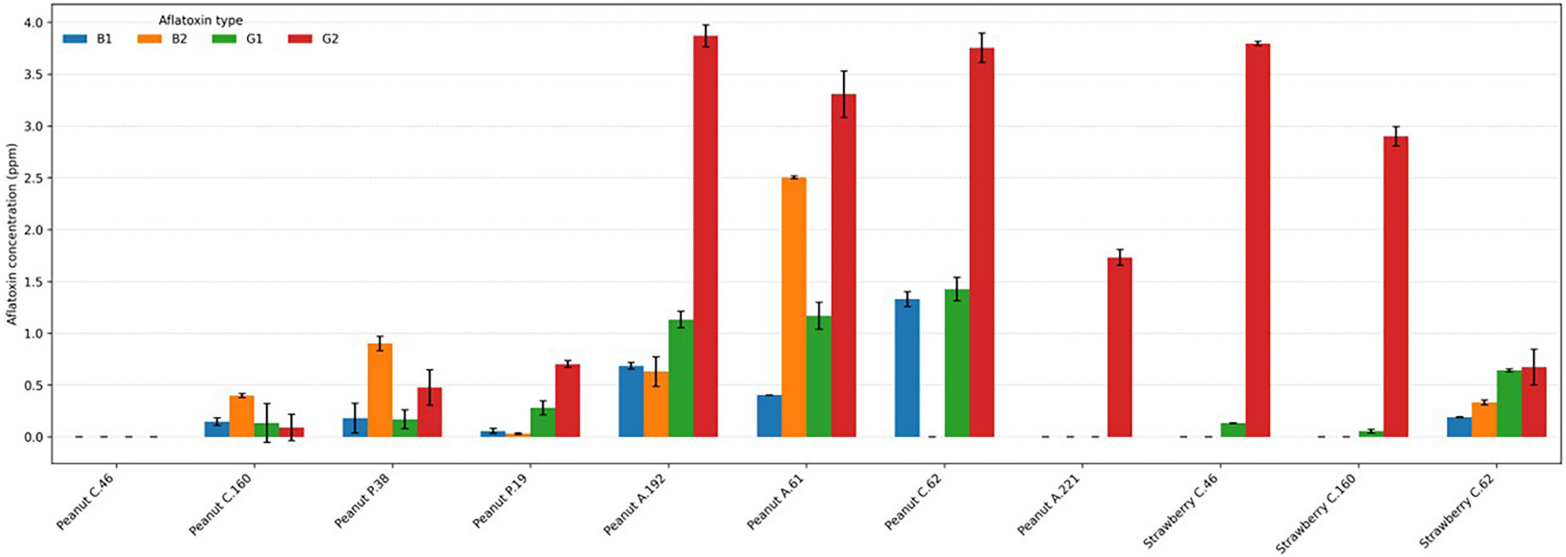
Aflatoxin production profiles of *A. quadrilineatus* strains grown on peanut and strawberry matrices as determined by HPLC analysis. Bars represent the mean concentrations (ppm) of aflatoxins B^1^, B^2^, G^1^, and G^2^ calculated from two technical replicates per sample, with error bars indicating standard deviation. Horizontal dashed gridlines are included to facilitate visual comparison of toxin levels across strains and food matrices.

Aflatoxin production varied markedly among fungal strains and between commodity matrices (peanut and strawberry). Quantification by HPLC revealed distinct toxin-specific profiles, with aflatoxins G^2^ and G^1^ generally dominating over B-type aflatoxins in several strains (Figure 5).

In peanut-derived isolates, strains A.61, A.192, and C.62 exhibited the highest overall aflatoxin concentrations (7.384, 6.317, and 6.511 ppm, respectively), characterized by pronounced G^2^ production accompanied by moderate to high levels of G^1^ and, in some cases, B-type aflatoxins. In contrast, strains C.46 and A.221 showed negligible or absent production of B^1^, B^2^, and G^1^, with A.221 producing G^2^ as the sole detectable toxin. In control samples, total aflatoxin levels did not exceed 4 ppb.

Isolates on strawberries displayed a different toxigenic pattern. Strains C.46 and C.160 produced minimal B-type aflatoxins but showed substantial accumulation of G^2^, whereas strain C.62 produced all four aflatoxins at moderate levels, with G-type toxins predominating.

One-sample *t*-tests were used to compare strain-specific aflatoxin production against the global mean concentration for each aflatoxin type (Supplementary Tables 3, 4). Several strains exhibited statistically elevated production prior to multiple-testing correction, particularly for G^2^ and B^2^ aflatoxins in peanut isolates and G-type aflatoxins in strawberry isolates.

After applying Benjamini–Hochberg false discovery rate (FDR) correction, only a subset of these effects remained statistically significant (*q* < 0.05; Supplementary Table 5). In peanut samples, strain A.61 showed significantly higher production of aflatoxins B^1^ and B^2^, while strain C.62 showed a significant elevation in aflatoxin B^1^ relative to the global mean. In strawberry samples, strain C.46 retained statistically significant overproduction of both aflatoxins G^1^ and G^2^ following FDR adjustment.

Other strains exhibited elevated toxin levels that did not remain significant after correction and were therefore considered non-significant trends rather than definitive differences against the global mean. Across both matrices, aflatoxin G^2^ emerged as the dominant toxin, accounting for the largest proportion of total aflatoxin burden in high-producing strains. This pattern was particularly evident in A.192 and A.61 (peanut) and C.46 (strawberry), where G^2^ concentrations exceeded those of B^1^, B^2^, and G^1^ by several fold. These results indicate strong strain-specific specialization toward G-type aflatoxin biosynthesis.

## 4 Discussion

Aflatoxin contamination of food and feed remains one of the most serious mycological threats to public health worldwide, particularly in regions characterized by high temperatures and fluctuating storage conditions. While classical aflatoxigenic species such as *Aspergillus flavus* and *A. parasiticus* have been extensively studied, comparatively little attention has been given to non-canonical species capable of producing sterigmatocystin and aflatoxins under favorable conditions. The present study provides compelling evidence that *Aspergillus quadrilineatus*, an indigenous member of *Aspergillus* section *Nidulantes*, represents an under-recognized food safety risk in arid environments.

All *A. quadrilineatus* isolates examined in this study exhibited optimal growth between 35 and 40°C, with several strains retaining growth above 45°C. This degree of thermotolerance is particularly relevant in arid and semi-arid regions such as Kuwait, where ambient temperatures frequently exceed 40°C during summer months and food storage environments often lack strict temperature control. The ability of these isolates to grow at temperatures commonly encountered during post-harvest handling and storage substantially increases the likelihood of persistence, colonization, and toxin production. Similar temperature-dependent growth patterns have been reported for other *Aspergillus* species, reinforcing the idea that climate plays a decisive role in shaping mycotoxigenic risk (Magan and Aldred, 2007; Baranyi et al., 2015).

Sporulation capacity on food substrates represents a critical determinant of fungal dissemination and secondary contamination. In this study, all isolates produced high conidial loads on infected peanuts and strawberries, with pronounced strain-dependent variability. Agricultural soil isolates generally exhibited higher sporulation than compost and poultry-associated strains, suggesting that repeated exposure to plant-derived substrates may select for enhanced reproductive fitness. Because fungal conidia are known to harbor and disseminate mycotoxins, elevated sporulation further amplifies the risk of food contamination and inhalation exposure (Sorenson et al., 1987; Dagenais and Keller, 2009). These findings underscore the importance of sporulation as both an ecological and toxicological trait.

Sterigmatocystin production was detected in all tested isolates, confirming that toxigenicity is a conserved feature of *A. quadrilineatus*. Peak sterigmatocystin accumulation occurred on day 7 of incubation, consistent with the established link between secondary metabolism and the transition from active growth to developmental differentiation in *Aspergillus* species (Calvo et al., 2002). Although sterigmatocystin is often considered less acutely toxic than aflatoxin B^1^, its carcinogenic properties and role as a biosynthetic precursor make its presence in food commodities a significant concern (Rank et al., 2011). Importantly, sterigmatocystin production did not strictly correlate with vegetative biomass, supporting previous observations that mycotoxin biosynthesis is regulated independently of fungal growth rate (O’Gorman, 2011).

One of the most striking findings of this study is the demonstration that multiple *A. quadrilineatus* isolates produced substantial quantities of aflatoxins on food substrates. Total aflatoxin concentrations in peanuts reached up to 7.38 ppm. Although such concentrations were achieved under optimal laboratory conditions, they nevertheless highlight the inherent biosynthetic capacity of this species. Importantly, the aflatoxin levels observed in *A. quadrilineatus* are comparable to those reported for the major aflatoxin-producing species *Aspergillus flavus* and *Aspergillus parasiticus*. Under laboratory or simulated storage conditions, *A. flavus* has been reported to produce total aflatoxin levels ranging from low μg/kg to several ppm, with values exceeding 5–10 ppm in highly toxigenic strains and conducive substrates, particularly peanuts and maize. Similarly, *A. parasiticus* is frequently associated with high aflatoxin production, often producing both B- and G-type aflatoxins at concentrations that can surpass several ppm under optimal conditions (Cotty and Jaime-Garcia, 2007; Frisvad et al., 2019; Kumar et al., 2021).

In contrast, many other *Aspergillus* species either lack the complete aflatoxin biosynthetic gene cluster or produce only trace amounts of aflatoxins, even under favorable conditions. The present findings therefore place *A. quadrilineatus* among a limited group of *Aspergillus* species with a pronounced capacity for aflatoxin biosynthesis. This suggests that its potential contribution to food contamination may be underestimated, particularly in warm and arid regions where environmental conditions favor fungal persistence and toxin production. The predominance of G-type aflatoxins, particularly aflatoxin G^2^, suggests strain-specific specialization within the aflatoxin gene cluster. Similar shifts in aflatoxin profiles have been reported in non-classical aflatoxigenic species and may reflect regulatory differences in pathway gene expression (Brown et al., 1996; Frisvad et al., 2004).

Substrate-dependent differences in toxin production were also evident. Peanuts supported higher total aflatoxin accumulation than strawberries, consistent with previous reports identifying oil-rich nuts as highly permissive substrates for aflatoxin biosynthesis (Tsitsigiannis et al., 2005). Nevertheless, the ability of *A. quadrilineatus* to colonize and intoxicate fresh produce such as strawberries is particularly concerning, as these commodities are often consumed raw and may bypass processing steps that reduce fungal load. The detection of aflatoxins in strawberries further expands the range of food matrices at risk from this species.

The ecological origins of the isolates examined in this study—agricultural soils, compost, and poultry farm environments—provide important insights into contamination pathways. Compost and agricultural amendments are increasingly recognized as reservoirs of mycotoxigenic fungi when not adequately treated (Barth et al., 2020). Our findings support a field-to-fork contamination model in which *A. quadrilineatus* can be introduced at multiple points along the food production chain, persist during storage, and ultimately reach consumers. In arid regions, where composting practices, dust exposure, and high temperatures intersect, this risk may be further amplified.

In addition to the experimental infection assays, we conducted a targeted screening to detect *Aspergillus quadrilineatus* in nuts purchased from retail markets in Kuwait using culture-based isolation approaches. Despite screening multiple samples, *A. quadrilineatus* was not recovered from market-derived nuts. This negative result likely reflects low prevalence in commercially available products, competitive exclusion by other storage-associated fungi, or effective post-harvest handling and storage practices rather than an inherent inability of the species to colonize nut substrates. Importantly, the absence of detection in retail samples does not diminish the biological relevance of our findings, as the controlled infection experiments clearly demonstrate the capacity of *A. quadrilineatus* to colonize nuts and produce high levels of mycotoxins under favorable conditions.

**FIGURE 6:**
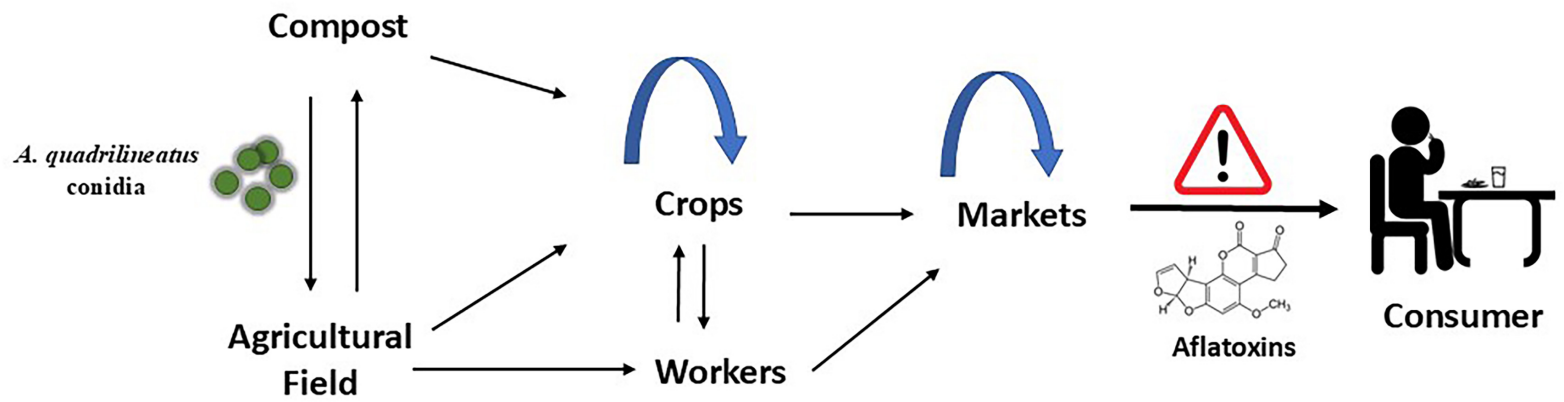
Conceptual framework illustrating the potential contamination routes through which *Aspergillus quadrilineatus* may reach the consumer’s table.

In conclusion, this study establishes *Aspergillus quadrilineatus* as a thermotolerant, sporulation-competent, and highly mycotoxigenic species capable of contaminating food commodities with sterigmatocystin and aflatoxins. These findings challenge the traditional focus on classical aflatoxigenic species and emphasize the need to broaden mycotoxin monitoring frameworks, particularly in arid and semi-arid regions. Improved surveillance, stricter compost management, and climate-aware food safety strategies will be essential to mitigate the emerging risks posed by non-canonical toxigenic *Aspergillus* species (Figure 6).

Future studies will extend this screening effort to locally grown produce, such as strawberries and palm dates, which are more directly exposed to indigenous agricultural soils, compost, and environmental dust. Integrating molecular detection methods alongside culture-based approaches will provide a more comprehensive assessment of the environmental distribution and food safety relevance of *A. quadrilineatus* in arid regions. Conduct genetic analysis of the aflatoxin biosynthesis pathway to explain the observed variability in toxin production among *A. quadrilineatus* strains. Targeted investigation of key genes within the aflatoxin biosynthetic cluster, together with whole-genome sequencing, would provide deeper insight into strain-specific aflatoxigenic potential and the genetic determinants underlying toxin biosynthesis.

## Data Availability

The datasets presented in this study can be found in online repositories. The names of the repository/repositories and accession number(s) can be found in the article/Supplementary material.
