# Supplementary material for: Aflatoxigenic potential of Aspergillus quadrilineatus: implications for food safety in arid climates
Source: Front Microbiol. 2026 Apr 30;17:1724920. doi: 10.3389/fmicb.2026.1724920 (PMC13171525; doi:10.3389/fmicb.2026.1724920)

**Supplementary Data**

Supplementary Table 1. The modified formulation from Borrow et al. (1961) typically includes:

| **Component** | **Quantity** | **Unit** |
| --- | --- | --- |
| Sucrose | 30 | g |
| Amino acids R 7131 (Sigma) | 3.2 | ml |
| Ammonium tartrate | 2.4 | g |
| Potassium dihydrogen phosphate (KH₂PO₄) | 5 | g |
| Magnesium sulphate heptahydrate (MgSO₄·7H₂O) | 1 | g |
| Special trace metal solution | 2 | ml |
| Agar | 20 | g |
| Distilled water | 1 | L |

Supplementary Table 2. One-sample t-tests against the global mean spore concentration (7.46 × 10⁵ spores/mL).

| **Strain** | **Mean (×10⁵)** | **p-value vs global mean** | **Interpretation** |
| --- | --- | --- | --- |
| **A.61** | 13.23 | **0.0036** | **Significantly higher** |
| **A.221** | 13.13 | **0.0171** | **Significantly higher** |
| C.62 | 8.47 | 0.376 | Not significant |
| P.19 | 8.47 | 0.376 | Not significant |
| A.192 | 5.91 | **0.0312** | Significantly lower |
| C.160 | 5.72 | **0.0272** | Significantly lower |
| P.38 | 5.72 | **0.0272** | Significantly lower |
| C.46 | 3.23 | **0.0067** | Significantly lower |
| P.74 | 3.23 | **0.0067** | Significantly lower |

Supplementary Table 3. One-sample *t*-test results showing p-values for each strain × aflatoxin combination in peanut seed samples, assessing whether strain-specific mean aflatoxin concentrations differ from the global mean concentration of the corresponding aflatoxin calculated across all samples and matrices.

| **Strain** | **Aflatoxin** | **Mean (ppm)** | **p-value** | **Interpretation** |
| --- | --- | --- | --- | --- |
| **A.192** | B1 | 0.686 | **0.035** | Significant |
|  | B2 | 0.63 | 0.308 | NS |
|  | G1 | 1.131 | 0.054 | Marginal |
|  | G2 | 3.87 | **0.025** | Significant |
| **A.61** | B1 | 0.404 | **0.002** | Highly significant |
|  | B2 | 2.504 | **0.003** | Highly significant |
|  | G1 | 1.169 | 0.083 | NS |
|  | G2 | 3.307 | 0.073 | NS |
| **C.62** | B1 | 1.329 | **0.012** | Significant |
|  | B2 | 0 | NA | Not testable |
|  | G1 | 1.426 | **0.019** | Significant |
|  | G2 | 3.756 | **0.031** | Significant |
| **P.38** | B1 | 0.181 | 0.533 | NS |
|  | B2 | 0.902 | 0.067 | Marginal |
|  | G1 | 0.17 | 0.134 | NS |
|  | G2 | 0.477 | 0.052 | Marginal |
| **P.19** | B1 | 0.058 | 0.421 | NS |
|  | B2 | 0.031 | 0.487 | NS |
|  | G1 | 0.281 | 0.061 | Marginal |
|  | G2 | 0.702 | **0.041** | Significant |
| **C.160** | B1 | 0.146 | 0.13 | NS |
|  | B2 | 0.4 | 0.235 | NS |
|  | G1 | 0.133 | 0.241 | NS |
|  | G2 | 0.09 | 0.318 | NS |
| **A.221** | B1 | 0 | NA | Not testable |
|  | B2 | 0 | NA | Not testable |
|  | G1 | 0 | NA | Not testable |
|  | G2 | 1.732 | 0.164 | NS |

Supplementary Table 4. One-sample *t*-test results showing p-values for each strain × aflatoxin combination in strawberry samples, assessing whether strain-specific mean aflatoxin concentrations differ from the global mean concentration of the corresponding aflatoxin calculated across all samples and matrices.

| **Strain** | **Aflatoxin** | **Mean (ppm)** | **p-value** | **Interpretation** |
| --- | --- | --- | --- | --- |
| **C.46** | G1 | 0.132 | **0.0047** | Significant |
|  | G2 | 3.796 | **0.0053** | Significant |
| **C.160** | G1 | 0.056 | **0.0209** | Significant |
|  | G2 | 2.899 | **0.0436** | Significant |
| **C.62** | B1 | 0.19 | **0.027** | Significant |
|  | B2 | 0.333 | 0.101 | NS |
|  | G1 | 0.643 | **0.0362** | Significant |
|  | G2 | 0.674 | 0.061 | Marginal |

Supplementary Table 5. Benjamini–Hochberg false discovery rate (FDR) correction applied to your one-sample t-test p-values (α = 0.05), across all strain × aflatoxin tests with non-zero variance.

| **Matrix** | **Strain** | **Aflatoxin** | **p-value** | **q-value** | **Status** |
| --- | --- | --- | --- | --- | --- |
| peanut | **A.61** | **B1** | 0.0024 | **0.0265** | Significant |
| peanut | **A.61** | **B2** | 0.0029 | **0.0265** | Significant |
| peanut | **C.62** | **B1** | 0.012 | **0.048** | Significant |
| strawberry | **C.46** | **G1** | 0.0047 | **0.0265** | Significant |
| strawberry | **C.46** | **G2** | 0.0053 | **0.0265** | Significant |

Supplementary Figure 1. Scanning electron micrograph of a cleistothecium of *Aspergillus quadrilineatus*.


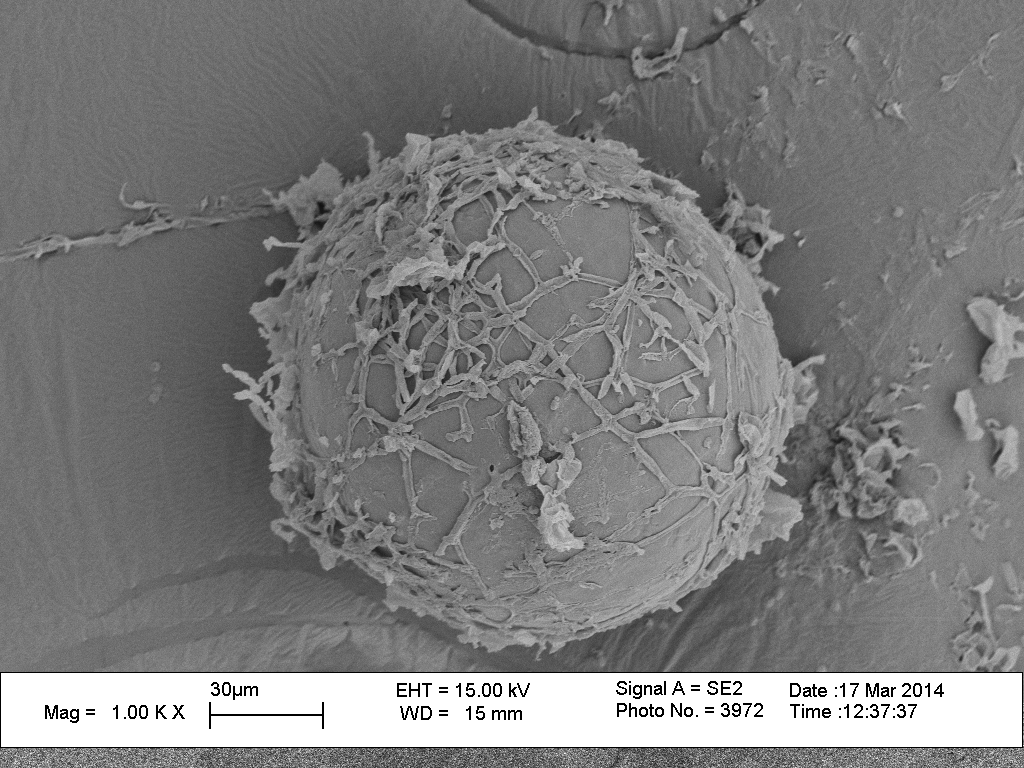

Supplement: Supplementary file 1 [file Supplementary_file_1.docx]
